# Replantation of Teeth

**Published:** 1873-10

**Authors:** 


*Replantation of Teeth.*—During the discussion of the subject of Alveolar Abscess at the late meeting of the Illinois State Dental Society, Dr. Chase, of Mo., remarked that these cases of replantation are more numerous than we should have supposed.

He said : " We know that teeth have been knocked out and lain in the dirt for two or three days, and after replacement have grown firm, and after recent luxation the circulation has become re-established.

In case of persistent alveolar abscess, he thought every one justified in conquering it by extraction and replantation.

It is astonishing what a tooth will bear. The idea that the periosteum is destroyed by cutting off the apex of the root is all a mistake. It is not at all necessary to keep the tooth in tepid water or blood. Time may be taken for the operation. In some cases it may be well, previous to operation, to take an impression for making a plastic retaining cap or splint upon, to be used after replacement of the tooth. He should treat the next case of a persistent nature that came under his care in this manner.

When there is no external opening in alveolar abscesses, forces creasote up ; it causes great pain.

Remembered a case, of two teeth treated in the same mouth, though not at the same time. One had an external opening, the other not. In the case without opening there was more pain than in the other, but it succeeded in a shorter time than the one that had the opening.

In a communication in a recent number of the *London Dental*

*Review*, the author advocates pepsine for dissolving dead portions of pulp. This struck the speaker favorably, and accordingly he had tried it for several months. It accomplished its work within a few hours, provided the pulp has not previously been treated with creasote or tannin "

Dr. Kilbourne, of Aurora, Ill., related a case of extraction of a first bicuspid, for incipient ulceration, and replantation. After cleaning root-canals with creasote, and filling with gutta percha, and filling carious cavity with tin, the tootl was replaced and left without ligation, and has been doing well since—about two years ago.

